# Measles Resurgence in Europe: An Open Breakthrough in the Field of Vaccine-Preventable Diseases

**DOI:** 10.3390/pathogens12101192

**Published:** 2023-09-25

**Authors:** Maria Antonia De Francesco

**Affiliations:** Institute of Microbiology, Department of Molecular and Translational Medicine, University of Brescia-Spedali Civili, 25123 Brescia, Italy; maria.defrancesco@unibs.it

Measles is a highly transmissible respiratory infection due to an enveloped, negative single-stranded RNA virus, belonging to the genus *Morbillivirus*, the family Paramyxoviridae and the subfamily Orthoparamyxovirinae [1].

After an incubation period that lasts 10–15 days, patients present a maculopapular exanthema that spreads starting from the face to the extremities. Generally, the subject is contagious from 4 days before the onset of symptoms to 4 days after the disappearance of the rash [2].

Generally, the infected patients recover quickly without adverse outcomes. However, complications associated with measles infection may be found in children younger than 5 years of age, in pregnant women and in immunocompromised subjects. Pneumonia, directly induced by the virus or as secondary infection, is one of the most frequent complications [3,4,5].

Neurologic complications are rarely observed, such as acute disseminated encephalomyelitis (ADEM) (1 case per 1000) that principally affects immunocompromised individuals [6,7,8]; and subacute sclerosing panencephalitis (SSPE) (6.5–11 cases per 100,000 patients), which occurs several years after the infection with the virus [9,10].

Laboratory diagnosis of measles is based both on serology to detect specific IgM antibodies in blood and on molecular biology to assay viral RNA presence in blood, urine, and respiratory specimens [11,12].

Measles is a vaccine-preventable disease, and its eradication is favored by many biological factors, including the presence of only one serotype, the genetic stability of the virus, the acquired lifelong immunity after the infection and the existence of only a natural viral host (humans). 

An effective vaccine was already introduced in 1963 [13]. The currently worldwide available vaccines are live attenuated vaccines, which are derived from the wild type Edmonston strain of the virus [14]. Vaccine formulations include the monovalent vaccine, bivalent (against measles and rubella, MR), trivalent (against measles, mumps and rubella, MMR), and tetravalent vaccines (against measles, mumps, rubella and varicella, MMRV) [15,16].

Two doses of the vaccine reach a 97% of efficacy in preventing measles, while one dose is 93% effective. Therefore, the World Health Organization (WHO) recommended two doses and a coverage of at least 95% to guarantee herd immunity for preventing outbreaks and for providing indirect protection for not vaccinated subjects [17].

Which vaccination programs for measles are present in the different European countries?

To date, MMR vaccination is recommended in 24 countries, and it is mandatory in 17 countries (Albania, Bosnia and Herzegovina, Bulgaria, Czech Republic, France, Hungary, Italy, Malta, Moldova, Montenegro, North Macedonia, Russia, Serbia, Slovakia, Slovenia, and Ukraine). In Germany, measles vaccination is mandatory, while vaccination for rubella and mumps is only recommended [18].

The final goal of the WHO was to implement vaccination plans in order to obtain a reduction in measles incidence and then the elimination of measles and of rubella and mumps worldwide [19,20,21,22].

Despite the introduction of vaccination programs in European countries, the increase in vaccine hesitancy and the absence of mandatory vaccination strategies in several countries generated the occurrence of different outbreaks throughout Europe [23]. 

In particular, in 2017, there were epidemics of measles in 28 European countries with 37 reported deaths. They were mostly detected in Romania (5608 cases), Italy (5098 cases), Greece (967 cases) and Germany (929 cases) [24]. Measles cases were also reported in European countries in 2018 (17,822 cases) and 2019 (13,199 cases) [25]. In 2020, measles decreased in all European countries with 2,043 cases due to the restrictions adopted during the COVID-19 pandemic, which was also responsible for a decrease in routine immunizations [25,26,27].

Between March 2021 and April 2022, the WHO reported 272 measles cases in Europe. Among them, 245 (90%) were detected in Tajikistan (97 cases), Turkey (57 cases), Belgium (15 cases), Poland (14 cases), Italy (13 cases), France (12 cases), Ukraine (12 cases), Germany (11 cases), Georgia (5 cases) and Russian Federation (5 cases) [28].

Between March 2022 and February 2023, the WHO updated the cases of measles (1861). Among them, 1728 (93%) were mostly found in ten countries: Tajikistan (610 cases), Turkey (466 cases), Russian Federation (414 cases), United Kingdom (67 cases), Serbia (40 cases), Austria (33 cases), Kyrgyzstan (29 cases), Poland (28 cases), France (22 cases) and Belgium (19 cases) [29].

The constant annual presence of measles cases in Europe highlights that measles eradication (reduction in measles incidence to zero) has still not been achieved and underlines the need to further increase adequate catch-up vaccinations for disease control and the prevention of new outbreaks.
